# Association between Postoperative Opioid Requirements and the Duration of Smoking Cessation in Male Smokers after Laparoscopic Distal Gastrectomy with Gastroduodenostomy

**DOI:** 10.1155/2021/1541748

**Published:** 2021-01-28

**Authors:** Chan-Sik Kim, Ji Hoon Sim, Yujin Kim, Seong-Soo Choi, Doo-Hwan Kim, Jeong-Gil Leem

**Affiliations:** Department of Anesthesiology and Pain Medicine, Asan Medical Center, University of Ulsan College of Medicine, Seoul 05505, Republic of Korea

## Abstract

Smoking is clinically associated with high postoperative pain scores and increased perioperative analgesic requirements. However, the association between the duration of smoking cessation and postoperative opioid requirements remains unclear. Therefore, this study aimed to evaluate the association between the duration of smoking cessation and postoperative opioid requirements. We retrospectively analyzed the data of 144 male patients who received intravenous patient-controlled analgesia (IV PCA) after laparoscopic distal gastrectomy with gastroduodenostomy. All patients were divided into three groups: G0, nonsmoker; G1, smoker who quit smoking within 1 month preoperatively; G2, smoker who quit smoking over 1 month preoperatively. Analgesic use, pain intensity, and IV PCA side effects were assessed up to postoperative day 2. As the duration of smoking cessation increased, the amount of postoperative opioid consumption decreased (*β* = −0.08; 95% confidence interval (CI), −0.11 to −0.04; *P* < 0.001). The total postoperative opioid requirements in G1 were significantly higher than those in G0 and G2 (G0, 75.5 ± 15.9 mg; G1, 94.6 ± 20.5 mg; and G2, 79.9 ± 19.4 mg (*P* < 0.001)). A multivariate regression analysis revealed that G1 was independently associated with increased postoperative opioid requirements (*β* = 12.80; 95% CI, 5.81–19.80; *P* < 0.001). Consequently, male patients who had ceased smoking within 1 month of undergoing a laparoscopic distal gastrectomy with gastroduodenostomy had higher postoperative opioid use than patients who had ceased smoking for more than 1 month and nonsmokers.

## 1. Introduction

Postoperative pain is one of the major causes of distress in patients during the postoperative period [1]. Given that inadequate postoperative pain management leads to increased morbidity, functional impairments, reduced quality of life, delayed recovery time, prolonged duration of opioid use, and higher healthcare costs [2, 3]; proper control of factors associated with increased postoperative pain is necessary. Recent studies have identified several factors associated with poorly controlled postoperative pain, and smoking was one of the major factors [4, 5].

Nicotine is considered to have an analgesic effect and smoking supplies nicotine to the central nervous system [6–8]. This nicotine activates both central and peripheral nicotinic acetylcholine receptors [9], which can enhance the descending inhibitory pain pathway, resulting in a potent analgesic effect even in postoperative pain [10]. However, available evidence suggests that smoking is clinically associated with high postoperative pain scores and increased perioperative analgesic requirements [5, 11–13].

One possible explanation for this could be the pathophysiologic changes that occur during smoking cessation, including nicotine withdrawal and altered nicotinic receptor availability [14–16]. These changes may develop after smoking cessation after prolonged exposure to smoking and persist for several weeks, which may be associated with increased pain sensitivity and postoperative opioid requirements [16]. Thus, a shorter duration of smoking cessation might be insufficient to recover these pathophysiologic changes, resulting in poorly controlled postoperative pain. However, in a detailed review of studies assessing the association between smoking and postoperative pain, the association between the duration of smoking cessation and postoperative opioid requirements was not adequately clarified [13]. Therefore, we evaluated the association between the duration of smoking cessation and postoperative opioid requirements in male patients who underwent a laparoscopic distal gastrectomy with gastroduodenostomy for early-stage stomach cancer.

## 2. Materials and Methods

### 2.1. Patients

**Figure 1:**
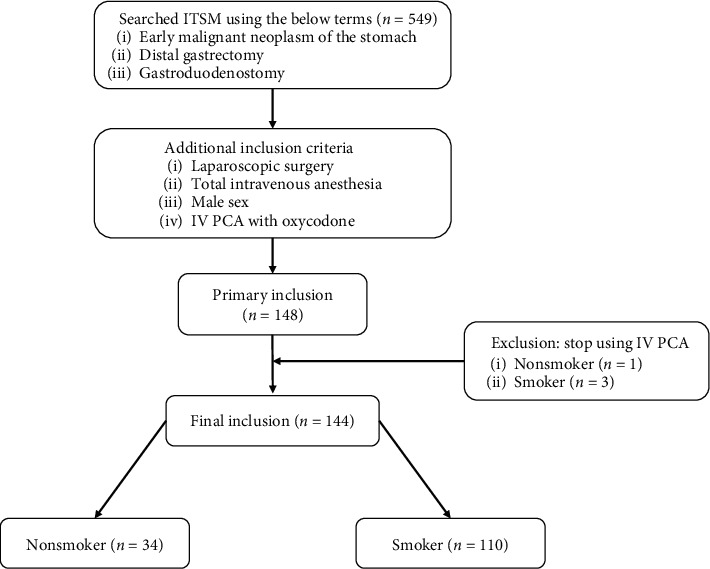
Flowchart of the present study. ITSM: Information Technology of Service Management; IV PCA: intravenous patient-controlled analgesia.

This retrospective study was approved by our institutional review board and registered at the University of Ulsan, Seoul, Republic of Korea (2020-0573). First, we searched our institution's Information Technology of Service Management (ITSM) system between September 2013 and February 2014 using the terms “early malignant neoplasm of the stomach,” “distal gastrectomy,” and “gastroduodenostomy.” Data on a total of 549 patients were retrieved after the first ITSM search. Further, patients undergoing laparoscopic surgery, patients receiving total intravenous anesthesia, male patients, and patients receiving intravenous patient-controlled analgesia (IV PCA) with oxycodone were included in the study. The rate of female smokers was less than 5% in this cohort study. Considering that the disproportionate smoking rates between sexes could be a confounding bias, women were excluded from our study. A total of 148 patients were thus eligible for inclusion after the second ITSM search was performed. A total of four patients who discontinued IV PCA because of dizziness, nausea, vomiting, or postoperative bleeding before the postoperative day (POD) 2 were also excluded. Finally, 144 patients (110 smokers and 34 nonsmokers) were included and analyzed (Figure 1).

Smoking status was defined based on a previous study's definition [17]. Nonsmokers were defined as individuals who had never smoked a cigarette or who had smoked fewer than 100 cigarettes in their lifetime. Current smokers were defined as individuals who had smoked more than 100 cigarettes in their lifetime and currently smoked. Ex-smokers were defined as individuals who had smoked more than 100 cigarettes in their lifetime but were not current smokers. In the present study, the smoker group included current smokers and ex-smokers. When scheduling the operation, current smokers were instructed to stop smoking before the surgery. All patients were divided into three groups: G0, nonsmokers, G1, smokers who had ceased smoking within 1 month of surgery, and G2, smokers who had ceased smoking more than 1 month before surgery.

### 2.2. Process of Anesthesia and Postoperative Analgesia

Total intravenous anesthesia was used for the induction and maintenance of anesthesia. To prevent severe postoperative pain after emergence, fentanyl (1 *µ*g/kg) or oxycodone (0.1 mg/kg) was injected 30 min before the end of the surgery. Before initiating IV PCA with oxycodone, 0.3 mg of IV ramosetron was administered to prevent postoperative nausea and vomiting. The IV PCA device (Accumate® 1100, Wooyoung Medical, Seoul, Korea) was set up with the following conditions: basal infusion rate, 1 mL/h; bolus dose, 1 mL; lockout interval, 15 min; and maximum infusion volume, 5 mL/h. The IV PCA regimen comprised oxycodone in 0.9% saline (total volume, 240 mL). The amount of oxycodone in the IV PCA was adjusted based on the patients' age and total body weight. Those who were aged <65 years received 200 mg if their total body weight was ≥50 kg and 160 mg if their body weight was <50 kg. Those who were aged ≥65 years and had a body weight ≥50 kg received 140 mg and received 100 mg if their body weight was <50 kg. After the surgery, when postoperative analgesia was considered insufficient (≥4 on the 11-point numeric rating scale), the patients received additional doses of fentanyl (1 *µ*g/kg) or oxycodone (0.1 mg/kg) in the postanesthesia care unit. For patients who experienced shivering, meperidine 25 mg was injected. In the general ward, rescue analgesics, including ketorolac 30 mg, tramadol 50 mg, and meperidine 25 mg, were administered to reduce postoperative pain. Ketorolac was initially administered when patients complained of significant postoperative pain (≥4 on the 11-point numeric rating scale). If the pain did not subside and the patients required additional analgesics, meperidine was administered. In patients with renal insufficiency, tramadol was initially administered instead of ketorolac to reduce postoperative pain.

### 2.3. Data Collection

The total dosage of analgesia required and pain intensity (11-point numeric rating scale [NRS] in which 0 = no pain and 10 = worst pain perceivable) were recorded daily up to POD 2. Nurses trained in the acute pain service evaluated all patients three times a day and recorded the amount of IV PCA solution administered and any side effects observed. The side effects of analgesics included nausea, vomiting, dizziness, pruritus, somnolence, and voiding difficulty. All opioids that were administered to patients were converted to IV oxycodone equianalgesic doses according to published conversion factors (IV fentanyl 100 *μ*g = meperidine 100 mg = oxycodone 10 mg) [18], and total postoperative opioid requirements were defined as the sum of daily opioid dosages for the first 2 days postoperation.

### 2.4. Statistical Analyses

Data were analyzed using the Statistical Package for the Social Sciences (SPSS) version 21.0 (SPSS Inc., Chicago, IL). All continuous data were tested for normality using the Kolmogorov–Smirnov test. The three groups' parametric continuous data were analyzed using one-way analysis of variance. The Kruskal–Wallis test was used to analyze nonparametric continuous data. Categorical data were compared using the chi-squared test or Fisher's exact test and presented as numbers and percentages. We performed regression analyses to evaluate the factors associated with postoperative opioid requirements. The most relevant factors after univariate linear regression analysis were compared through multiple regression analysis. Additionally, to assess changes in postoperative opioid requirements in smokers according to the duration of smoking cessation, linear regression analysis was performed. A *P* value <0.05 was considered statistically significant.

## 3. Results

The demographic characteristics of the three groups are shown in Table 1. There were no significant differences between the groups in terms of baseline characteristics, except the duration of smoking cessation.

**Figure 2:**
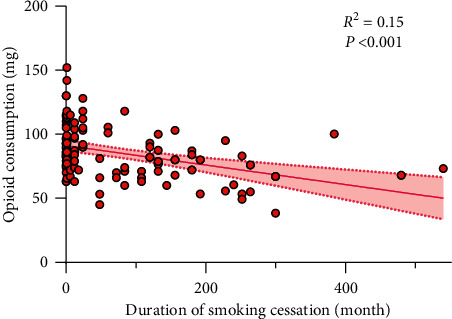
Scatterplot and regression line of opioid requirements according to the duration of smoking cessation. Pink zone between dotted lines indicates the 95% confidence interval.

Figure 2 presents the association between the duration of smoking cessation and total postoperative opioid requirements (for the G1 and G2 groups alone). As shown below, as the duration of smoking cessation increased, the amount of postoperative opioid requirements decreased (*β* = −0.08; 95% confidence interval (CI), −0.11 to −0.04; *P* < 0.001).

**Figure 3:**
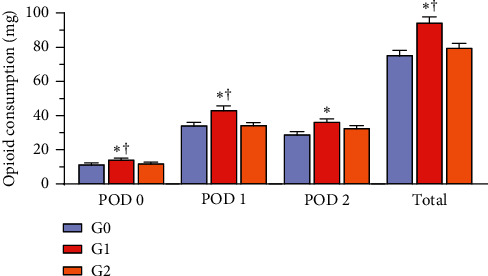
Comparison of opioid requirements between the three groups. ^*∗*^*P* value <0.05 compared with G0 †*P* value <0.05 compared with G2. There were no significant differences between G0 and G2. Data were analyzed using one-way analysis of variance. PODs 0, 1, and 2 = postoperative days 0, 1, and 2, respectively.

The postoperative opioid requirements at PODs 0 and 1 were significantly higher in G1 than in G0 or G2 (POD 0: G1, 14.6 ± 3.1; G0, 11.8 ± 3.6 (*P*=0.004); G2, 12.3 ± 4.1 mg (*P*=0.006) and POD 1: G1, 43.4 ± 15.2; G0, 34.5 ± 9.0 (*P*=0.003); G2, 34.7 ± 10.0 mg (*P* < 0.001)). Similarly, the total postoperative opioid requirements were significantly higher in G1 than in G0 or G2 (total: G1, 94.6 ± 20.5; G0, 75.5 ± 15.9 (*P* < 0.001); G2, 79.9 ± 19.4 mg (*P* < 0.001)) (Figure 3).

On POD 2, the postoperative opioid requirements in G1 were significantly higher than those in G0 (36.6 ± 9.9 mg vs. 29.2 ± 7.9 mg, respectively; *P*=0.004) and there was no significant difference between G1 and G2 (36.6 ± 9.9 mg vs. 32.9 ± 10.4 mg, respectively; *P*=0.138).

The demand for rescue nonsteroidal anti-inflammatory drugs was also significantly higher in G1 than in G0 and G2 on POD 1 (G0, 1.0 [0.0–1.0]; G1, 1.0 [1.0–2.0]; G2, 1.0 [1.0–1.0]; *P*=0.005). On POD 2, the demand was not significantly different between the three groups (*P*=0.420).

**Figure 4:**
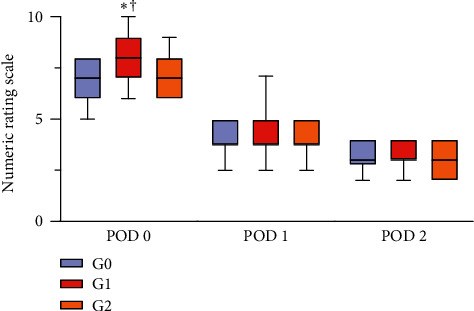
Comparison of numeric rating scale scores between the three groups according to postoperative day ^*∗*^*P* value <0.05 compared with G0. †*P* value <0.05 compared with G2 PODs 0, 1, and 2 = postoperative days 0, 1, and 2, respectively. There was no significant difference between G0 and G2. The horizontal line inside the boxes shows the median values; the upper and lower edges of the box represent the third and first quartiles, respectively. The whiskers above and below the boxes represent 90% and 10%, respectively. Data were analyzed using the Kruskal–Wallis test.

On POD 0, the NRS score in G1 was significantly higher than that in G0 or G2 (G0, 7.0 [6.0–8.0]; G1, 8.0 [7.0–9.0]; G2, 7.0 [6.0–8.0]; *P* < 0.001) (Figure 4). On PODs 1 and 2, there was no significant difference in NRS scores between the three groups.

Univariate linear regression analysis revealed that age and G1 were associated with increased postoperative opioid requirements after laparoscopic distal gastrectomy with gastroduodenostomy (age: *β* = −0.62, 95% CI [−0.90 to −0.34], *P* < 0.001; G1: *β* = 16.22; 95% CI: [9.31–23.13]; *P* < 0.001) (Table 2).

These relevant factors associated with postoperative opioid requirements after the univariate linear regression analysis were included in the multivariate linear regression analysis. As a result, age and G1 were independently associated with postoperative opioid requirements (age: *β* = −0.46, 95% CI [−0.74 to −0.17], *P*=0.001; G1: *β* = 12.80, 95% CI [5.81–19.80], *P* < 0.001). The side effects recorded up to POD 2 were not different between the three groups (Table 3).

## 4. Discussion

The current study presented three main findings. First, postoperative opioid requirements decreased in accordance with an increased duration of smoking cessation in patients who were smokers. Second, male patients who ceased smoking within 1 month preoperatively had more postoperative opioid requirements and showed higher pain intensity than nonsmokers and patients who ceased smoking more than 1 month preoperatively. Third, younger age and smoking cessation within 1 month of surgery were independently associated with increased postoperative opioid requirements.

Several previous studies have demonstrated that opioid requirements and pain intensity increased in smoker patients during the postoperative period [5, 14, 19]. Some studies have also found an association between the amount of smoking and postoperative opioid requirements [5, 20]. However, to the best of our knowledge, a report assessing the quantitative change of postoperative opioid requirements according to an increased duration of smoking cessation in smokers has not yet been presented. In the present study, we showed that postoperative opioid requirements significantly decreased in accordance with an increased duration of smoking cessation in smoker patients. Based on this finding, we further evaluated the postoperative opioid requirements according to a specific duration of smoking cessation.

Altered pathophysiologic changes as a result of smoking cessation have been well established. For example, significant improvements in blood pressure and heart rate are observed within 24 hours of smoking cessation, lung function improves within 3 months, and the risk of coronary heart disease decreases to half after 1 year of smoking cessation [21, 22]. Although these associations are well established, the specific duration of smoking cessation that leads to improved postoperative pain outcomes has not been defined. Recently, Zhao et al. [13] evaluated the effect of smoking cessation before surgery on postoperative pain and analgesic requirements after thoracoscopic radical resection of lung cancer in elderly male patients. The authors found that patients who ceased smoking more than 3 weeks before surgery had lower postoperative pain scores and lower postoperative opioid requirements than those who ceased smoking less than 3 weeks prior. Our findings are consistent with the findings of that study. Smoking cessation within 1 month of surgery showed higher postoperative opioid requirements up to POD 2 than smoking cessation over 1 month before surgery and nonsmoking. Postoperative pain scores were also higher for those who had stopped smoking within 1 month of surgery than for those who had stopped smoking over 1 month before surgery and nonsmokers.

These findings may be explained, at least in part, by nicotine withdrawal and altered nicotinic receptor availability [14–16]. Smoking-related chronic nicotine exposure can induce nicotine dependence and withdrawal [8]. Withdrawal symptoms, including depressed mood, anxiety, insomnia, and irritability, may develop in abstinent smokers and can lead to increased pain sensitivity and opioid requirements postoperatively [14, 16]. In addition, in acutely abstinent smokers, *β*2 nicotinic acetylcholine receptor availability is increased, which is associated with increased pain sensitivity [15]. These pathophysiologic changes may begin within a few hours of abstinence from nicotine and can be sustained for several weeks [8]. Therefore, several weeks of smoking cessation may be required to reverse these pathophysiologic changes. Smoking cessation within 3 or 4 weeks of surgery appeared to be insufficient to reduce pain perception and opioid requirements postoperatively, based on our results and previous findings [13]. In the present study, smokers in the early stage of smoking cessation had increased postoperative opioid requirements. Consequently, increased postoperative analgesia or multimodal analgesia may be required to provide sufficient pain management in patients who cease smoking within 1 month of surgery because adequate analgesia is important to achieve favorable postoperative outcomes [8, 23].

Younger age is well known to be a risk factor for increased pain intensity and opioid requirements postoperatively [20, 24]. The present study and our previous study have conﬁrmed that age is an independent factor associated with postoperative opioid requirements [5]. Moreover, in this study, smoking cessation within 1 month was independently associated with increased postoperative opioid requirements. Although the results of a previous study assessing the duration of smoking cessation and postoperative opioid requirements are consistent with the findings of the present study [13], the causality between the duration of smoking cessation and postoperative opioid requirements could not be determined. In the present study, a multiple regression analysis was performed to adjust for confounding bias. Therefore, we believe that smoking cessation within 1 month was associated with increased postoperative opioid requirements. Consequently, this study can be helpful to establish the guidelines for proper postoperative pain management and to determine the duration of abstinence required for smokers undergoing surgery.

This present study has some limitations. First, this was a retrospective study comprising a small sample size, which could limit and weaken the results. However, we performed various statistical analyses, including multivariate linear regression analysis. Hence, we believe that these methods strengthened the statistical significance of our results. Second, we included not only current smokers but also ex-smokers. In this study, current smokers had ceased smoking to achieve better surgical outcomes, at least 1 week or a maximum of 6 weeks before the surgery, and they were almost included in G1, except for one patient who was classified as G2 because the duration of smoking cessation was 6 weeks. However, ex-smokers ceased smoking to reduce their risk for smoking-related disease or healthcare costs, at least 6 months or a maximum of 45 years before the surgery, and they were all included in G2. Thus, the duration of smoking cessation for the ex-smokers was longer than that for the current smokers. These differences may have affected the pathophysiologic changes observed as a result of smoking cessation subsequently. Consequently, further evaluation of current or ex-smokers only is warranted. Third, although we used 1 month of smoking cessation as the criterion when dividing smokers into two groups, further study with various durations of smoking cessation to reduce postoperative pain is necessary. Fourth, although subjects with chronic pain or those who were taking pain medication were not considered for exclusion when designing the present study, through the strict review of medical records, we confirmed no patients who had chronic pain or used any pain medication for chronic pain in our cohort.

## 5. Conclusions

In conclusion, male patients who had ceased smoking within 1 month of undergoing a laparoscopic distal gastrectomy with gastroduodenostomy had higher opioid requirements than male patients who had ceased smoking over 1 month before surgery and nonsmokers. Postoperative opioid use appeared to be reduced according to an increased duration of smoking cessation in smokers. After adjusting for other clinical variables, smoking cessation within 1 month was considered an independent factor associated with increased postoperative opioid use. Therefore, more postoperative analgesics may be required to provide sufficient analgesia in patients who cease smoking within 1 month of surgery.

## Figures and Tables

**Table 1:** Patients' characteristics.

Group	Nonsmokers	Smokers		*P* value
	G0 (*N* = 34)	G1 (*N* = 42)	G2 (*N* = 68)	
Duration of smoking cessation (months)		0.2 (0.2–1.0)	108.0 (24.0–180.0)	<0.001
Age (years)	59.9 ± 10.8	52.7 ± 9.9	60.1 ± 11.5	0.444
BMI (kg/m^2^)	24.2 ± 2.3	24.0 ± 2.6	23.9 ± 2.4	0.537
Pack-year	0	27.6 ± 14.5	22.6 ± 14.5	0.084
Duration of anesthesia (min)	139.9 ± 14.5	143.6 ± 24.6	145.0 ± 19.1	0.237
Intraoperative remifentanil consumption (mg)	2.8 ± 0.6	3.1 ± 0.8	2.8 ± 0.8	0.938

Data are expressed as mean ± standard deviation or median (interquartile range). Pack-year was calculated by multiplying the number of packs of cigarettes smoked per day with the number of years the person has smoked. BMI: body mass index; G0: nonsmokers; G1: smokers with ≤1 month of cessation preoperatively; G2: smokers with >1 month of cessation preoperatively.

**Table 2:** Linear regression analysis of factors associated with the increased postoperative opioid requirement.

Parameters	Univariate			Multivariate		
	*β*	95% CI	*P* value	*β*	95% CI	*P* value
Age (years)	−0.62	−0.90 to −0.34	<0.001	−0.46	−0.74 to −0.17	0.001
Groups classified by the duration of smoking cessation						
G0 (nonsmoker; reference)	0			0		
G1 (≤1 month)	16.22	9.31–23.13	<0.001	12.80	5.81–19.80	<0.001
G2 (>1 month)	−6.23	−12.92–0.46	0.068			
BMI (kg/m^2^)	0.54	−0.87–1.95	0.449			
Anesthesia duration (min)	−0.14	−0.31–0.03	0.106			
Intraoperative remifentanil consumption (mg)	3.62	−0.69–7.92	0.099			

*β* indicates regression coefficient; CI: confidence interval; BMI: body mass index; G0: nonsmokers, G1: smokers with ≤1 month smoking cessation preoperatively; G2: smokers with >1 month smoking cessation preoperatively.

**Table 3:** Opioid side effects up to postoperative day 2.

Group	Nonsmokers	Smokers		*P* value
	G0 (*N* = 34)	G1 (*N* = 42)	G2 (*N* = 68)	
Nausea/vomiting	3 (8.8%)	1 (2.4%)	5 (7.4%)	0.450
Dizziness/somnolence	2 (5.9%)	2 (4.8%)	5 (7.4%)	0.857
Urinary retention	2 (5.9%)	0 (0.0%)	3 (4.4%)	0.320

Data are represented as the number of cases (percentage). G0: nonsmokers; G1: smokers with ≤1 month smoking cessation preoperatively; G2: smokers with >1 month smoking cessation preoperatively.

## Data Availability

The datasets generated and/or analyzed during the current study are available from the corresponding author on reasonable request.
